# A 10-week judo-based exercise programme improves physical functions such as balance, strength and falling techniques in working age adults

**DOI:** 10.1186/s12889-021-10775-z

**Published:** 2021-04-17

**Authors:** Marina Arkkukangas, Karin Strömqvist Bååthe, Anna Ekholm, Michail Tonkonogi

**Affiliations:** 1 Research and Development in Sörmland, Eskilstuna, Sweden; 2 Department of Physiotherapy, School of Health, Care and Social Welfare, Mälardalen University, Västerås, Sweden; 3 Department of Medicine, Sport and Fitness Science, School of Education, Health and Social Studies, Högskolan Dalarna, Falun, Sweden

**Keywords:** Judo, Fall prevention, Fall accidents, Workplace, Physical exercises

## Abstract

**Background:**

Falls and fall-related injuries are major threats not only for older adults but also for younger age groups such as working-age adults. It has been shown that it is possible to reduce the risk of falls and fall-related injuries, to some extent. However, interventions aiming at reducing both the risk of falls and mitigating fall-related injuries through teaching safe falling techniques are still sparsely investigated. The aim with this study was to investigate the effect of a 10-week workplace-based judo inspired exercise programme (Judo4Balance). The measures in the study include physical functions, fall-related self-efficacy and safe falling techniques.

**Methods:**

A total of 142 working-age adults participated in this non-randomised controlled study. The participants were allocated to the Judo4Balance group (*n* = 79), or to a waiting list control group (*n* = 63). The mean age was 47 years (18–68). The recruitment period was from May 2018 to October 2019. A total of 128 participants were included in the analysis. Logistic Regression models were used to analyse the outcomes: physical function, balance and fall-related self-efficacy as well as falling techniques (backwards and forwards).

**Results:**

At the 10-week follow-up, the results displayed significant differences between the two groups in all measurements, except for the fall-related self-efficacy with OR = 1.8. Techniques for falling forwards and backwards displayed the highest OR = 124.1 and OR = 98.9. Physical function and balance showed OR = 3.3 and OR = 6.4.

**Conclusions:**

This exercise programme under study displayed significant differences in strength, balance and safe falling techniques between the groups. It is suggested that these functions, which were studied here, can effectively be trained in working-age adults by using the Judo4Balace exercise programme. Thus, it may be beneficial to further investigate and include training in proper falling techniques when designing fall prevention exercise programmes. Furthermore, it may be a novel way of addressing fall-related injuries, which are of utmost importance to prevent in near future.

**Trial registration:**

NCT04294342. Registered 4 March 2020 - The Impact of Specifically Adapted Judo-based Training Program on Risk Factors for Falls Among Adults - Full Text View - ClinicalTrials.gov

## Background

Falls are a major occurrence and common threat to older adult’s health worldwide [1]. In six EU nations (France, Germany, Italy, Spain, Sweden and UK), approximately 2.7 million fractures occur annually in those aged 50 or above due to falls. These accidents lead to a healthcare cost of €37 billion, and by 2030 the cost is predicted to increase by 23% [2].

Falls have been suggested as being the leading cause of non-fatal injuries treated in hospital emergency departments in almost all ages, except in the age group of 15–24 years [3]. In addition, ground level unintentional falls are the most common work incident, with approximately 8000 of these falls occurring at work annually in Sweden [4]. In the US, slips, trips and falls among school district employees have been reported to have the highest frequency of injuries at work [5].

The risk of falling increases with age also at work. Women in the age category 56–64 years have a 2.4 times higher risk of experiencing a ground level unintentional fall at work compared to the average woman at work. For men, the corresponding age group has a 1.8 times higher risk of experiencing ground level unintentional falls at work compared to the average man at work. This relationship between age and the risk of falling in the working population suggests that similar to older adults, the decline in physical ability represents a major risk factor for falls [4].

In Sweden, 11,000 reported accident insurance claims yearly relate to falling accidents, resulting in more than 30 days of sick leave and/or a degree of permanent disablement. Fractures are the most common injury, leading to a permanent disability [4]. The most common injury after a fall at ground level is fractures in the upper limbs, because the person tries to break the fall with an arm or hand, corresponding to 42% of all fractures in men and 50% of all fractures in women. This is followed by injuries to the lower limbs, corresponding to 33% in men and 28% in women; then follows injuries to the core, hips and lower back. Multiple injuries are rather rare, representing only 1% of fall injuries at work in both men and women. Although head injuries only represent 3% of the total number of injuries of the ground level falls, they can lead to very serious consequences [4].

A fall in older age groups is almost always a consequence of declining physical function, which, in turn, often leads to loss of independence, fear of falling and low fall-related self-efficacy [6, 7]. Evidence shows that exercises targeting strength and balance reduce falls and fear of falling among older adults [8, 9], indicating exercises to be one of the most important fall prevention actions. Besides known associations between strength balance deficits and falls, one overlooked aspect is being able to or learning to get up and down from the floor and falling safely [10]. Therefore, in a long-term perspective, exercise habits targeting muscle strength, balance, stability and safe falling strategies developed and maintained throughout the entire lifespan help to reduce accidents at work and also have an impact on future risk factors for falls [11]. In Sweden, many employers are offering their employees a free hour of exercise per week during work hours called “health hour”. Many larger employers also offer gym time and exercise classes during lunch breaks or in connection with the start and end of the working day.

Given the lack of knowledge about exercises targeting risk factors for falls in conjunction with learning falling techniques among working-age adults, one suggestion has been to explore judo exercises as such an exercise form. Judo is a popular sport worldwide and the level of physical fitness, tactical skills and techniques required in judo is high [12, 13]. Since known fall prevention actions mainly include strength and balance exercises, learning falling techniques (including getting up and down from the floor) could be an additional way of combating risk factors for falls and fall-related injuries in the future. It has been shown previously that learning landing strategies, which is one aspect of falling safely, can significantly reduce impact load during a fall and might be effective in reducing the risk of injury from a fall [14].

A specific Judo4Balace programme was developed 2017 in Sweden by a, in judo, black belted task force composed of three physiotherapists, one occupational therapist, a medical doctor supported with scientific knowledge and a programme review from staff at the University of Dalarna. A black belt in judo indicates that the instructor has been trained in judo for at least 10 years and is skilled in breakfall techniques in all directions. The Judo4Balance programme includes exercises aimed at improving strength, balance and safe falling techniques (so-called “breakfalls” in martial arts terminology). To our knowledge, there are no previous studies investigating the effects of performing specific exercise at work, aiming at reducing known risk factors for falls among working-age adults. To address this group, it is imperative to explore exercises provided in a workplace setting, as previous studies have successfully conducted interventions aiming to promote physical activity at the workplace [15].

Therefore, the aim of this study was to investigate the effects of a 10-week workplace-based judo inspired exercise programme targeting physical functions such as strength and balance, fall-related self-efficacy and falling techniques compared to a control group.

## Method

This study was a non-randomised controlled trial with one intervention group and one control group. The study included 142 participants from various workplaces in Sweden, including 79 participants in the intervention group and 63 in the control group.

### Setting

Workplaces representing construction and steel industry, healthcare, pre-school, insurance office, advertising, and health and safety consultants showed an interest in participating in the study. These workplaces were located in different regions of Sweden. An announcement/offer was made through the insurance company’s website and through social media. Workplaces that had reported fall-related injuries to the insurance company in the insurance database during last few years were contacted by email. We strived to get a good mix of workplaces where employees had sustained fall-related accidents. Workplaces representing industries with the highest identified fall incidence were chosen for the project. First, the workplace management agreed to arrange an exercise group at the workplace and agreed to carry out follow up research. The workplace then announced the possibility for employees to participate in the Judo4Balance programme. Once the employee agreed and signed up for the exercise program each individual participant was offered to participate in the study. The participants got a written information as well as verbal information and then signed their letter of consent.

After getting their acceptance to participate and written consent was obtained, the participants were directed to one of the two groups. The study used a convenience sampling where the first persons to accept participation were directed to the intervention group. If the work schedule did not permit participation at the planned time point, the person was put on a waiting list e.g. control group, and was offered the training programme at a later date when follow-up measures were completed and the study was closed. The average age of the participants was 48 (SD 11.3) and 45 (SD 12.2) years in the control and intervention group, respectively. The gender distribution was 75/25% (women/men) in the control groups and 61/39% (women/men) in the intervention group.

Inclusion criteria were being over 18 years of age, ability to understand written and verbal Swedish language and being available at the workplace for the testing and training.

Exclusion criteria included uncontrollable high blood pressure or retinal detachment, which does not allow for exercise.

A total of 14 qualified judo4Balance instructors led the different exercise groups (on average, two instructors per group). The participants in the control group received the intervention after completing the measurements at the 10-week follow-up. All measurements were taken at the participant’s workplace and were conducted by three trained test leaders.

### Intervention

The 10-week intervention included 10 sessions (50 min /week), as previously described in Arkkukangas et al. [16]. Briefly, each session included a warmup, balance and coordination exercises, breakfall training, strength training and a short cool down Furthermore, there were some more high intensity judo inspired exercises and games, such as trying to touch each other’s feet, rolling and crawling on the mat, and simple gymnastics aimed at increasing proprioception (perception and awareness of the position and movement of the body).

All judo instructors were well acquainted with the Judo4Balance programme as well as experienced in leading group exercises. To increase participant’s motivation, many of the exercises were performed in pairs (a common practice in judo). Breakfall training comprises the traditional four main “breakfalls” taught in judo: Backward falling, sideways breakfall (both right and left), forward breakfall without a roll, and forward breakfall with a roll. The 10-week programme was planned with a progression, divided into three blocks.

1) Practicing basic falling techniques such as falling backwards and sideways from a sitting on your buttocks or kneeling position and strength exercises, body awareness, basic balance and mobility training, including getting up and down from the floor.

2) Continuing with falling techniques, increasing load in strength exercises, challenging balance and coordination ability, greater range of movements and possibly power in strength exercises.

3) Continuing with advanced falling techniques (for example, from standing up position), challenging balance and strength exercises and ability to develop power in strength exercises.

### Measurements

The measurements in this study are commonly used to evaluate physical functions, balance and fall-related self-efficacy for older adults. Therefore, these well-known instruments have been recently expanded and adapted for this specific target group [16]. Additionally, physical activity level was evaluated before and after the intervention. Three specially trained test leaders performed the measurements pre- and postintervention. Pre-test were conducted within 1–3 weeks before the intervention. Postintervention measurements were performed within 1–3 weeks.

*Physical performance* in this study was measured by the Short Physical Performance Battery (SPPB) [17], which assesses physical performance in the lower extremities. SPPB was expanded with additional progression including advanced items in balance (tandem with heel raise, and tandem with heel raise with closed eyes), gait speed (backwards), and lower body power (chair stand on one leg -left/right) [16]. The original scale scores range from 0 to 4, with a total score of 12 points. The total score with the additional items is 32 points. A total score of 32 represents the best performance. In adults over the age of 60 the original SPPB has shown to predict the risk of falls and has shown to have good test-retest reliability [17].

*Balance* was measured by the Mini-BESTest [18], which includes 14 different tasks on four subscales, with a total score of 28 points and has shown good test-retest reliability and inter-rater reliability [19]. The extended test was expanded with additional progression by adding six items [16]. All tasks are graded from 0 to 2 points, with a total maximum score of 40 points for the extended test.

*Fall-related self-efficacy* e.g. self confidence in the ability to perform various daily activities without falling was measured by Falls Efficacy Scale-Swedish version (FES-S) [20]. FES-S has previously shown high test-retest reliability [21]. The FES-S has been extended from 13 items to 19 items with six additional questions [16]. Each item is rated from 0 to 10, with a maximum score of 190 points, which represents the highest level of self-efficacy.

*Activity level*- this measure was registered at baseline to describe the participants’ activity habits from study start. In addition, the activity level was evaluated postintervention. The Frändin/Grimby activity scale [22] was used, where activity level is estimated on a 6-point scale. The scale considers activities typically performed during the winter and summer seasons, with the addition of workplace setting in this study; higher score represents higher activity level. The scale has shown to be valid for assessing physical activity among older adults [22].

I this study the letter “W” was added to the initial abbreviation for measures used to indicate that extended versions adapted for working age participants were applied. All participants performed the tests according to the original manual; thereafter, the extension was added. The expanded instruments were pilot tested in 12 healthy volunteers in different ages (10–95 years).

Since there are no previously known tests for the evaluation of falling techniques, two new tests were recently developed by Arkkukangas et al. [16] to test the skills acquired for falling backwards as well as falling forwards in a safe setting. Briefly, the judo fall techniques were graded on a 0–4 scale. Not being able to lay down and raise the head from the floor and then stand up resulted in zero points and falling safely from a standing position resulted in four points, which represents the best performance. The first 80 tests were rated by at least two test leaders, well experienced in judo teaching and evaluating breakfall technique.

A power analysis has been performed on SPPB-W data. To be able to detect significant differences corresponding to three arbitrary units given α-value of 0.05 and β-value of 0.80, a total of 82 participants (41 in each group) is required. Since the study involved 142 participants, the data has sufficient statistical power.

### Data analysis

Baseline characteristics for the exercise group and the control group are presented as proportion (gender), mean (age) and median (all tests). Between groups analysis were performed by using chi-square test, t-test and Mann Whitney U test. Whitin group analysis were performed by using Wilcoxon Signed Ranks Test. Per protocol analysis was applied; no data were imputed.

Logistic regression was used to estimate associations between the groups and changes in test results from baseline to follow-up. Logistic regression analysis was conducted individually for every parameter analysed (FES-S-W, SPPB-W, Mini-BEST-W, falling techniques backwards and forwards). Age and gender are factors known to influence the parameters investigated. Therefore, the logistic regression analysis was adjusted for age and gender.

The results are reported as odds ratios (OR), 95% confidence intervals (95% CI) and *p*-values with a significance level of 0.05. All analyses were performed using the statistical programme SPSS 22.0 for Windows (SPSS Inc., Chicago, IL).

## Results

The study was conducted from May 2018 to November 2019. The results of the 142 participants in the two groups are presented in Table 1. The reasons for the 10% dropout (142 at baseline and 128 at follow-up) were, not being available for follow-up measurements because of or change in workplace or being on sick leave during the actual day of the test. Mean value of adherence was 7.5 out of the 10 sessions. No adverse events were reported. No significant differences were detected between the groups regarding age, gender, or any other parameters at baseline.

**Table 1:** Test values (median and (min-max)) of the participants at baseline (*n* = 142), follow up (*n* = 128) and difference within groups and between groups

Outcomes	Controls		Exercise	
	Baseline *n* = 63	Follow-up *n* = 59	Baseline *n* = 79	Follow-up *n* = 69
Activity level Summer (1–6)	4 (1–6)	4 (1–6)	4 (2–6)	4 (3–6) *
Activity level Winter (1–6)	4 (1–6)	4 (1–6)	4 (2–6)	4 (2–6)
Activity level Workplace (1–6)	3 (1–6)	3.5 (1–6)	3 (1–6)	4 (1–6) **
FES-S-W (0–190)	175 (106–190)	176 (37–190)	179.5 (87–190)	181 (128–190) *#
SPPBW-W (0–32)	21.5 (12–30)	22 (10–30)	23 (9–30)	26 (12–31) **#
Mini-BEST-W (0–40)	31 (17–37)	30 (20–39) *	31 (14–36)	33 (24–37) **##
Falling techniques backwards (0–4)	1 (1–4)	1 (1–4)	1 (0–4)	4 (1–4) **##
Falling techniques forwards (0–4)	1 (1–3)	1 (1–3)	1 (1–3)	3 (1–4) **##

*Note.* FES-S-W = Falls Efficacy Scale- Swedish version-Working age, SPPB-W = Short Physical Performance Battery-Working age, MiniBEST-W = Mini-BESTest- Working age, n = numbers

* indicates a significant difference within group (* *p* < 0.05, ** *p* < 0.001)

# indicates a significant difference between groups (# *p* < 0.05, ## *p* < 0.001)

The range in proportions of improvements in each group displayed 55–90% for the exercise group and 7–49% for the control group. Falling techniques displayed a total of 81% (90–9%, 88–7%) difference between the exercise and control groups on both measures.

A total of 128 participants out of 142 were included in the regression models, which were adjusted for age and gender, with 69 participants in the exercise group and 59 in the control group. The results display significant differences between the groups in all measurements, except for fall-related self-efficacy with an OR of 1.8 and 95% CI = 0.9–3.7. The techniques for falling forwards displayed the highest OR = 124.1 (95% CI = 32.2–478.2) and backwards OR = 98.9 (95% CI = 28.5–342.8), see Table 2.

**Table 2:** Logistic regression models adjusted for age and gender, exercise group *n* = 69 and control group *n* = 59

Outcomes	*OR*	*95% CI*	*P*
FES-S- W	1.8	0.9–3.7	0.12
SPPB-W	3.3	1.5–7.0	**< .005**
Mini-BEST-W	6.4	2.9–14.0	**< .001**
Falling techniques- Backwards	98.9	28.5–342.8	**< .001**
Falling techniques- Forwards	124.1	32.2–478.2	**< .001**

*Note.* FES-S-W = Falls Efficacy Scale- Swedish version-Working age, SPPB-W = Short Physical Performance Battery-Working age, MiniBEST = Mini-BESTest- Working age, *OR* = Odds Ratio, *CI* = Confidence Intervals *P* = *p*-value, *N* = Numbers, bold figures indicate a significant value ≤0.05

## Discussion

The results in this study confirm the exercise intervention, Judo4Balance, to be effective, with significant differences between the intervention and control group in physical functions and falling techniques. However, the study showed no significant differences between the groups in fall-related self-efficacy in the logistic regression model. Targeted risk factors in this study (strength and balance) are known functions closely related to falls [23, 24]. Sherrington et al. reported in 2017 and 2019 [23, 24] that balance exercises should be challenging and involve three or more hours/week of exercise. However, this study confirms less than one hour (50 min) per week to be sufficient to gain significant improvements on physical functions, including balance, which, in turn, is an important risk factor for falls. Gawler et al. (2016) also suggested that exercise programmes for active older adults should be shorter, more challenging and more rapidly progressive, to be effective [25]. In addition, it has been shown that resistance training is associated with balance recovery [26], which is important in fall prevention and therefore included in our study.

The results herein displayed significant improvements in falling techniques with high OR 98.9–124.2 for learning techniques for falling backwards and forwards. These results add to those from a previous study by Groen et al., which reported reduction in impact forces after a short training period of falling techniques among older adults [27]. However, in the study by Groen et al., falling techniques were performed from a kneeling position [27], whereas in our study falling techniques were, when appropriate, performed from a standing position. These results should be highlighted and are important to include in further research in fall prevention, as it is of utmost importance to decrease and prevent fall-related injuries, especially fractures [28]. In this study, we used a newly developed instrument for measurements of falling techniques. Our research team plans to further test and validate this instrument since, to our knowledge, there are no available validated measures for specific falling techniques. Until then, the results should be interpreted with some caution.

In our study, we included the measure of fall-related self-efficacy as it is evident from previous research that exercises have been shown to increase fall-related self-efficacy and fear of falling, known to be major risk factors for falls [29–31]. However, the results in our study did not confirm such results, which may indicate that fall-related self-efficacy is an age-related concern, and is also related to an older and more sedentary population [32]. This adds to the body of literature demonstrating the importance of individually tailored interventions.

Conducting the intervention in a workplace setting also leads to a discussion about addressing health inequities and inequalities in health promotion policies [33]. Some health inequalities are unavoidable, whereas others can be influenced or changed and addressed; in this study, by offering training at work. Thus, achieving participation and adherence to physical activity and exercise in the broad population is known to be challenging. Studies have highlighted the social context and supervision to be crucial for achieving both participation and adherence [34]. Participation in activities, especially social, and physical leisure activities decreases with age, in both men and women [35]. Previous study shows that a decrease in these activities is closely related to increased risk for falls [6]. The participants in our study were considered as having high activity levels from baseline with a median value of 3–4 on Frändin/Grimby activity scale (1–6 points) [22]. This strengthens the discussion of the intervention being effective, since this active sample of study participants displayed significant results, although they already had a high activity level. To gain significant improvements in an already active sample requires more effective exercise programmes. Sedentary behaviour and time spent sitting still at work have increased in recent decades, especially among young and middle-aged groups. Inactivity and long periods of sitting are closely related to increased risk of obesity, heart disease, and premature death, and research have so far failed to present effective interventions [35]. Since the physical functions increased at the 10-week follow-up, this study could be used in preventive efforts in workplace settings when aiming to promote health among workers. This study shows that it may be possible for working-age adults to acquire falling techniques. Thus, it may be beneficial to include the effects of training proper falling techniques in further fall prevention research and in work exercise programmes.

### Limitations

This study was a non-randomised controlled trial, which may be seen as a limitation; however, there were no significant differences between the groups considering age, activity level and gender at baseline. This study targeted a broad public, with a wide age range, different occupations and education levels, and varying physical training status, which could be viewed as a limitation as well as a strength. Delivering an intervention suitable for all ages regardless of age or gender is considered a strength in this study, as it more accurately represents an actual workplace composition of employees. Since this study reports significant positive results from the intervention, these findings could be useful for replication in future studies. We also performed a peer protocol analysis, which may be contraindicated to use in clinical trials. The decision to exclude intention-to-treat analysis (ITT) was based on arguments of uncertainty regarding true efficacy of treatment and using ITT, providing heterogeneity in the sample, and an increased risk of type II error [36]. There was a dropout both in the intervention and in the control group. However, the dropout rate was low and is unlikely to have affected the results.

## Conclusion

The results in this study confirm the exercise intervention, Judo4Balance, to be effective in improving physical functions and falling techniques compared to a control group in working age adults. Improvements in falling techniques should especially be highlighted since this function could be an important tool for avoiding or mitigating the severity of fall-related injuries. These results are recommended for inclusion in further fall prevention research and should also be investigated from a long-term perspective. The Judo4Balance programme had no impact on fall-related self-efficacy in this study sample of active adults, which should be further investigated. We believe that our study contributes significantly to the literature because it adds to the body of knowledge on what types of exercises appeal to a broad public, for example, at the workplace, and points out important physical functions and falling techniques that need to be further investigated in relation to the outcome of falls.

## Data Availability

The datasets generated and/or analysed during the current study are not publicly available but are available upon request to the corresponding author.

## Abbreviations

SPPB: Short physical performance Battery
FES(S): Falls Efficacy Scale- Swedish version
OR: Odds Ratio
CI: Confidence Interval
